# Corticosteroids for Acute and Recurrent Idiopathic Pericarditis: Unexpected Evidences

**DOI:** 10.1155/2019/1348364

**Published:** 2019-12-16

**Authors:** Antonio Perrone, Anna Castrovilli, Giuseppina Piazzolla, Sabina Savino, Alessia D'Introno, Carlo Sabbà

**Affiliations:** Interdisciplinary Department of Medicine, Internal Medicine Unit “Cesare Frugoni”, School of Medicine, University of Bari, Piazza G. Cesare 11, 70100 Bari, Italy

## Abstract

Pericarditis is a common disease, often postviral or “idiopathic,” diagnosed in about 5% of emergency room visits for non-ischemic chest pain. Although pericarditis often occurs as a benign and self-limiting disease, it may present recurrences. The first-line therapy includes aspirin/nonsteroidal anti-inflammatory drugs (ASA/NSAIDs) *plus* colchicine. Steroids especially at high-dose have been associated with a higher recurrence rate. In this retrospective study, we evaluated efficacy and safety of ASA/NSAIDs and prednisone in the treatment of acute or recurrent idiopathic pericarditis (colchicine was off-label in the period of the study). The cohort included 276 patients diagnosed with acute idiopathic pericarditis. Mean age was 45.4 ± 12.7 years, and males were significantly higher in number and younger than females. Sixty-one patients (22.1%) were treated with prednisone and 215 with ASA/NSAIDs (77.9%). 171 patients experienced at least one recurrence (62%). No difference in recurrence rate was observed (*p*=0.257) between the groups treated with prednisone (55.7%) vs. ASA/NSAIDs (63.7%). The recurrences were treated with steroids at low doses and very gradual tapering, and the dose reduction was slower as the number of relapses was higher. Steroids alone were administered to about 80% of patients, while in the remaining 20% of cases, they were associated with ASA/NSDAIDs or colchicine. Approximately 90% of patients had a very favorable course, that is no more than 2 relapses and no patients presented serious side effects. Steroids at low dose, did not act, surprisingly, as an independent risk factor for recurrences and therefore may be considered a successful and safe treatment for acute and recurrent idiopathic pericarditis.

## 1. Introduction

Acute pericarditis (AP), with or without effusion, is the most common pericardial disease, it accounts approximately for 0.2% of all cardiovascular admission [1], and 5% of patients visited in an Emergency Department for nonischemic cardiac chest pain [2].

It occurs more often in men aged 20 to 50 years; however, the prevalence data in elderly subjects (>65 years) are poor due to the limited studies [3].

There are multiple causes of AP which can be broadly divided into infectious and noninfectious [4–6], but most of AP (80–90%) are of unknown etiology, labeled as idiopathic [7–9].

The AP prognosis is generally good, and recurrences are reported after 15–30% of all acute idiopathic pericarditis [3, 10]. It is supposed an occult autoimmune etiology for these recurrent subtypes [10, 11]. In a few cases, severe complications, such as cardiac tamponade and constrictive pericarditis [12, 13], may occur and they are usually related to the etiology of pericarditis.

The treatment of AP is largely empirical except those cases with a specific etiology. Medical therapy includes nonsteroidal anti-inflammatory drugs (NSAIDs), aspirin (ASA), colchicine, and steroids. ASA/NSAIDs *plus* colchicine represents the first line of idiopathic pericarditis treatment [10]. Steroids are recommended in those patients with contraindications or failure of ASA/NSAIDs *plus* colchicine [10] as they are considered an independent risk factor for recurrent pericarditis [10, 14].

However, some issues remain unclear about the therapy of acute idiopathic pericarditis (AIP), i.e., what is the best duration of therapy, what are the best doses, how best tapering steroids, and why the association steroid-colchicine does not reduce and may even increase the AP recurrence rate [15].

The aim of the present study was to evaluate the efficacy and safety of ASA/NSAIDs and steroids for the treatment of acute or recurrent idiopathic pericarditis.

## 2. Materials and Methods

This study is a retrospective analysis of a cohort of patients admitted to the Internal Medicine Unit “Cesare Frugoni” of Bari University Hospital with a diagnosis of AP during the time period of January 1993 to December 2016.

The diagnosis of AP was based on at least two of the following criteria: (a) typical pericardial chest pain, (b) pericardial friction rubs upon auscultation, (c) characteristic electrocardiogram (ECG) changes, and (d) new pericardial effusion mainly detected by echocardiography [10].

We descriptively analysed demographic parameters, clinical presentation, physical examination findings, laboratory (creatinine, electrolytes, troponin I, liver enzymes, CRP, haemoglobin, leucocytes, thyroid hormones, antinuclear antibodies, anti-DNA antibodies, rheumatoid-factor, serum electrophoresis, urine analysis, viral and neoplastic markers, and Mantoux or QuantiFERON-TB Gold test) and instrumental (ECG, chest X-ray, echocardiogram, chest CT/MRI scan) investigations, underlying etiology, comorbidities, and therapies. In no case, HIV test and pericardial biopsy were performed.

These clinical investigations allowed classifying AP into idiopathic and secondary to a known etiology.

Pericardial effusion was evaluated by echocardiography and classified as a mild (<10 mm, estimated volume <200 ml), moderate (10–20 mm, estimated volume 200–500 ml), and large effusion (>20 mm, estimated volume >500 ml) [16, 17]. Pericardiocentesis and the subsequent analysis of pericardial fluid were performed only in patients with cardiac tamponade and hemodynamic impairment. Follow-up was performed only in AIP patients, re-evaluating clinical and laboratory features approximately every 3 months. The median follow-up time was 23.5 months (range 11–36 months).

Pericarditis relapse was attained according to the same clinical criteria utilized for diagnosis of acute pericarditis. A diagnosis of recurrent pericarditis was made if the relapse occurred after 4–6 or more weeks symptom-free interval, during therapy discontinuation or pharmacological tapering [10].

### 2.1. Statistical Analysis

Continuous data were reported as mean ± SD; categorical variables were reported as frequency and percentage. Patient groups were compared by use of Student's *t* test for continuous variables and *χ*^2^ analysis or Fisher's exact test, as appropriate, for categorical variables. A value of *p* < 0.05 was considered statistically significant.

## 3. Results and Discussion

### 3.1. Clinical Presentation and Baseline Features

During the study period, 313 cases of AP (1.2% of the annual hospitalized patients) were recorded. The patients were more likely to be male (214; 68.4%) than female (99; 31.6%) with mean age of 45 ± 12.6 years (range 17–76 years).

Diagnosis of AIP was made in 276 of 313 patients (88.2%).

Among 37 patients with pericarditis of known aetiology (11.8%), metastatic neoplasms were found in 40.5% of cases, 8 cases developed AP after cardiac surgery (21.6%), a diagnosis of autoimmune disease was made in 7 patients (18.9%), 3 subjects had post-traumatic pericarditis (8.1%), tuberculosis pericarditis was diagnosed in 2 cases (5.4%), and uremic pericarditis was shown in 2 patients with end-stage renal disease. Detailed features of patients with a pericarditis secondary to a known aetiology are reported in Table 1.

Table 2 describes demographic, clinical and laboratory characteristics of AIP patients. Males were significantly more numerous than females (67.8%, M/F: 2.1, *p* < 0.001), mean age 45.4 ± 12.7 years. However, when the subjects were stratified by age, the sex-based differences in prevalence were statistical significant only in the group aged ≤65 years (M/F: 2.3, *p*=0.002), while no differences were detected in the group aged >65 years (M/F ratio = 0.6, *p*=0.78). Male patients were significantly younger than female (43.5 ± 11.8 yrs vs. 49.7 ± 13.3 yrs, *p* < 0.001). The most prevalent presenting symptom was chest pain (98.6%), followed by cough (43%) and dyspnea (18%). On physical examination, pericardial rub was detected in 104 patients (38%), and fever was present on admission in 102 cases (37%). Typical ECG changes were found in 204 subjects (74%). Pericardial effusion on echocardiography was shown in 182 subjects (66%) and was estimated as severe in 44 patients (24%), moderate in 51 patients (28%), and mild in 87 patients (48%). Pleural effusion was observed in 96 subjects (35%). Cardiac tamponade was detected in 11 patients, and in 7 patients with hemodynamic compromise, an emergency pericardiocentesis was performed, showing in all cases a serohematic fluid. Only in those cases, pericardial fluid analysis was carried out and did not yield a specific diagnosis in any case. Pericardial biopsy was not performed. White blood cells, C-reactive protein (CRP), and erythrocyte sedimentation rate (ESR) elevations were observed in almost all patients as reported in Table 2.

### 3.2. Patient Management

As shown in Table 3, ASA/NSAIDs were administered to 215/276 patients with AIP (78%), in particular ASA (1.5–3 g daily) to 194/276 patients (70.2%) and NSAIDs, namely, ibuprofen (1.2–1.8 g daily), to 21/276 patients (7.6%). Corticosteroids (prednisone) were prescribed in 61/276 patients (22.1% of cases) after the exclusion of tubercular or purulent pericarditis, at variable doses (0.25–0.5 mg/kg/daily, until the resolution of symptoms and normalization of the inflammatory markers, generally for two-three weeks, followed by gradual tapering of 5 mg each two-three weeks). Patients treated with corticosteroids were significant older (57 ± 7.1 vs. 42 ± 12 *p* < 0.001) and presented more relevant pericardial effusion (44 vs. 0, *p* < 0.001), dyspnea (36.1% *vs* 12.6), and TnI (41% *vs* 24%) than ASA/NSAIDs-treated patients. Moreover steroids were used more frequently in patients affected by diseases that requested their use as haemolytic autoimmune anemia, De Quervain thyroiditis (5 vs. 0, *p* < 0.001).

### 3.3. Follow-Up Data

After a median follow-up of 23.5 months (range, 11–36 months), recurrences of pericarditis were diagnosed in 171 AIP patients (62%) and they were more common in ASA/NSAIDs-treated patients than in the ones treated with corticosteroids (64% *vs* 56%); however, the difference was not statistically significant (*p*=0.257) (Table 4).

As shown in Table 5, among patients with recurrent pericarditis, 109 patients (63.8%) had one episode, 43 patients (25.1%) had 2 episodes, 18 patients (10.5%) had three to four episodes, and only one patient had five episodes.

Despite the recommendations of ESC 2015 guidelines [10], in Italy, until April 2017, colchicine was off-label for the AIP treatment; therefore, it was prescribed in a small number of patients of this study; in particular, it was added (off-label) to corticosteroids in 13 patients who experienced ≥3 episodes, whereas the other patients were treated with prednisone alone or in combination with ASA or NSAIDs, except for the patient who had 5 relapses and was successfully treated with IVIG.

No cases of deaths, severe side effects (heart failure, osteoporosis, myopathies, neurological disorders, and bleedings, severe progression of renal disease), or steroid dependent recurrent pericarditis were recorded.

## 4. Discussion

Currently available diagnostic tests (hematochemical, serological, cultural, and histological examination on biopsies) in most cases do not allow to establish the exact etiology of AP that therefore is named acute idiopathic pericarditis (AIP).

The high prevalence of AIP patients observed in our study (Table 1) is consistent to that reported from other studies [4–6].

As shown in Table 1, the neoplastic pericarditis prevailed among the secondary forms and in particular those associated with melanoma that are rarely highlighted in the literature [18]. Neoplastic pericarditis are frequently associated with cardiac tamponade, as shown in this study.

Table 2 shows age and sex significant differences that were found in AIP subjects. The observed sex differences are unknown, although a role of male sex hormones can be supposed, as suggested by some experimental studies on myocarditis in mice [19, 20]. It seems that testosterone may play an important role by the inhibition of anti-inflammatory cells [21], the increase of viral binding to the myocytes [22], and the stimulation of immune responses mediated by Th1-lymphocytes [20].

In our study, the mean age at onset of AIP was lower in male subjects compared to that in women. However, when the patients were stratified by age, the difference of mean age at clinical onset between male and female subjects was observed only in the younger age group (<65 years), while the gender distribution was nearly balanced in the patients aged >65 years. These results agreed with those reported in another study, where the male predominance was much more pronounced in patients younger than 45 [23].

Compared with the literature, clinical, laboratory, and instrumental features were equally frequent.

As shown in Table 3, in first line, about 80% of patients were treated with aspirin or NSAIDs while for the remaining 20% steroids, at variable doses and variable tapering, were used.

Steroids were generally administered to elderly, to those patients with more severe clinical features, comorbidities that needed steroids (autoimmune haemolytic anemia, thyroiditis, *etc.*), contraindications to or intolerance of ASA/NSAIDs.

No other differences in clinical variables were found between patients treated with ASA/NSAIDs vs. prednisone. As for steroids, high doses (until 2 mg/kg/day) were used in the most severe forms (associated with myocarditis or severe pericardial effusion). In the literature, although corticosteroids produce a fast and satisfactory clinical response, their use has been identified as an independent risk factor for recurrence based on observational studies [12, 24–30]. Because most cases of AIP are presumed to be postviral, it is believed that corticosteroids causing immunosuppression may impair antiviral immune response. Patients treated with high-dose steroids showed higher rate of side effects and a higher rate of relapses and hospitalizations [25]. So it could be believed that low-dose steroids are better, but the data are still limited and controversial. Currently, the recommended dose of prednisone is 0.25 to 0.5 mg/kg/day for 2–4 weeks, followed by a slow tapering [10, 14, 25].

Table 4 shows that the rate of first recurrences was more common in ASA/NSAIDs-treated patients (63.7%) than in patients treated with corticosteroids (55.7%), but the difference was not statistically significant (*p* value = 0.257). This unexpected result shows the following: (i) same efficacy of low-dose steroids when used as first-line therapy, compared to ASA/NSAIDs, since they determined not statistically different recurrence rates; (ii) steroids, in this study, were not an independent risk factor for recurrences, unlike literature statements [10, 14].

In the literature, the frequency of first recurrences varies from different studies from 20% and 30% [24, 31], while the recurrence rate after the first relapse varies between 20% and 50% especially in those patients not treated with colchicine or after corticosteroids therapy [32, 33]. In this study, the patients treated with ASA/NSAIDs or steroids had both a first recurrence rate higher than the one reported in the literature (Table 4) [6, 34, 35]. This high rate of relapse is probably related to (a) the absence of colchicine as first-line therapy (in Italy, colchicine was off-label until April 2017) and (b) a “selection bias,” as our clinic, in Southern Italy, was considered a reference centre for the most severe or resistant-to-therapy AIPs. Many patients arrived directly to our department with very severe clinical manifestation, while others were treated, at first line, in different hospitals and transferred to our department for the severity of clinical manifestations or resistance to the treatment. Unfortunately, many of them presented a history of inappropriate first-line therapy (low doses of ASA/NSAIDs or too short duration of the therapy).

A limitation of this study is that the number of patients treated in the first line with steroids alone is considerably lower than the number of patients treated with ASA/NSAIDs; therefore, it is not possible to express a definitive judgment on the rate of first recurrences.

As shown in Table 5, all recurrences were treated with steroids. Steroids alone were administered in about 80% of patients, while in the remaining 20% of cases, they were associated with ASA/NSDAIDs or colchicine. Recurrences were treated with low-dose steroids and very gradual tapering and the dose reduction was much slower as the number of relapses was higher. As a result of this recurrence treatment scheme, approximately 90% of patients had a very favorable course, that is no more than 2 relapses. In addition to this result, no patients presented serious side effects (i.e., myopathies, neurological/psychiatric disorders, osteoporosis, and dispeptic gastric disorders), and in no cases, preventive treatments were administrated. Patients who, instead, had a less favorable prognosis (≥3 recurrences) constituted only 10% of the total. By evaluating first-line therapies, these subjects had a too rapid steroid tapering. In these subjects, colchicine was added to the steroids. All of these patients had no more recurrences except only one (5 recurrences). This was a young patient diagnosed with recurrent pericarditis associated with multiple sclerosis. The use of intravenous immunoglobulins (IVIG) for the fifth recurrence treatment definitively interrupted the pericarditis recurrences and resolved the symptomatology of the multiple sclerosis.

Based on the results of this retrospective study and our clinical experience, we suggest that prednisone 0.25–0.5 mg/kg/day given until the resolution of symptoms and normalization of the inflammatory markers, generally for two-three weeks, followed by gradual tapering of 5 mg each two-three weeks (based on the clinical and laboratory response), and further 2.5 mg each two-three weeks, until complete suspension, is a successful and safe treatment for AIP.

In resistant forms, multidrug therapy should be considered, adding ASA or NSAIDs or colchicine to steroids, even if the optimal treatment has not yet been established. The treatment with immunosuppressive agents (azathioprine, cyclosporine, cyclophosphamide, and methotrexate) [36, 37], IVIG, anakinra [38] (IL-1 receptor antagonist), and canakinumab [39] (IL-1*β* blocking monoclonal antibody), may be considered only in cases of recurrent pericarditis which is refractory or intolerant to conventional treatments, but data for their use are limited, and should be tailored to the specific individual patients. Finally, pericardiectomy may be performed for frequent and highly symptomatic recurrences of pericarditis, which is resistant to medical treatment.

## 5. Conclusions

In our study, prednisone showed an equal efficacy compared to ASA/NSAIDs, having determined the same first recurrence rate, as it was not really an independent risk factor for recurrences, unlike the literature evidences [10, 14].

The recurrence rate after the first line treatment was, in the entire study population, higher than the expected on the basis of literature data, probably due to the absence of colchicine in first line and a selection bias of the patients.

The treatment of recurrent pericarditis showed the efficacy of steroids at low doses and very gradual tapering, resulting in a notable reduction of recurrence rate and a low percentage of prolonged courses, unlike the literature evidences [10, 14].

Steroids, at low doses and gradual tapering, were, also particularly safe as no serious side effects occurred without any preventive therapy (i.e., vitamin D), while ASA/NSAIDs at high doses, for prolonged time, may get worse gastric dyspeptic disease, renal failure, arterial hypertension, and heart failure, especially in elderly.

The AIP therapy must be tailored on the phenotype of the patient, considering important variables such as severity of the clinical manifestations, age, comorbidities, and side effects of therapies and interactions between the pharmacological treatments.

Therefore, based on the results presented, although in the literature there are discordant evidences and opinions not favorable to the use of steroids for the first line treatment of idiopathic pericarditis [10, 14], we believe that they may be considered safe and effective when administered at low doses and very gradual tapering, especially in the current “aging era,” in which many more patients will not be able to tolerate high-dose NSAIDs.

## Figures and Tables

**Table 1:** Etiology of acute pericarditis in the overall study population.

Etiology	Incidence
Idiopathic, *n* (%)	276 (88.2)
Secondary	37 (11.8)
Infective, *n* (%)	2 (0.6)
Autoimmune, *n* (%)	7 (2.2)
SLE, *n* (%)	3 (1)
Undifferentiated connectivitis, *n* (%)	2 (0.6)
Still disease, *n* (%)	1 (0.3)
Horton disease, *n* (%)	1 (0.3)
Neoplastic, *n* (%)	15 (4.8)
Lung, *n* (%)	5 (1.6)
Lymphoma, *n* (%)	3 (1)
Melanoma, *n* (%)	3 (1)
Breast, *n* (%)	2 (0.6)
Myeloma, *n* (%)	1 (0.3)
Gastric, *n* (%)	1 (0.3)
ESRD, *n* (%)	2 (0.6)
Post-surgery, *n* (%)	8 (2.6)
Post-trauma, *n* (%)	3 (1)

Values are *n* (%). SLE indicates systemic erythematous lupus; ESRD, end-stage renal disease.

**Table 2:** Demographic and clinical data of the 276 patients with acute idiopathic pericarditis.

Feature	Value
Age, *y* (mean ± SD)	45.4 ± 12.7
Male	43.5 ± 11.8^*∗*^
Female	49.7 ± 13.3^*∗*^
Male, *n* (%)	187 (67.8)^*∗*^
Thoracic pain, *n* (%)	272 (98.6)
Fever, *n* (%)	102 (36.9)
Cough, *n* (%)	118 (42.8)
Dyspnea, *n* (%)	49 (17.8)
Upper respiratory tract infections in the previous 2 weeks, *n* (%)	149 (53.9)
Diarrhea, *n* (%)	35 (12.7)
Pericardial rub, *n* (%)	104 (37.7)
Pleural effusion, *n* (%)	96 (34.8)
Pericardial effusion, *n* (%)	182 (65.9)
Severe	44 (24.2)
Moderate	51 (28)
Mild	87 (47.8)
Cardiac tamponade, *n* (%)	11 (3.9)
Emergency pericardiocentesis *n*, (%)	7 (2.5)
ECG typical changes, *n* (%)	204 (73.9)
WBC > 10^3^/mm^3^, *n* (%)	240 (87.7)
CRP > 2.9 mg/L, *n* (%)	273 (98.9)
cTnI > 0.045 ng/ml, *n* (%)	77 (27.8)
ESR > 20 mm/h, *n* (%)	266 (96.4)

Values are mean ± SD or *n* (%). ECG indicates electrocardiogram; WBC, white blood cells; CRP, C-reactive protein; cTnI, cardiac troponin I; ESR, erythrocyte sedimentation rate. ^*∗*^*p* value < 0.001.

**Table 3:** Baseline clinical characteristics of AIP patients treated with prednisone *vs* ASA/FANS.

Feature	Prednisone (61)	ASA/FANS (215)	*p* value^*∗*^
Age, *y*	57 ± 7.1	42 ± 12	<0.001
Female, *n* (%)	22 (36.1)	67 (31.2)	
Thoracic pain, *n* (%)	59 (96.7)	213 (99.1)	
Fever, *n* (%)	21 (34.4)	81 (37.7)	
Cough, *n* (%)	27 (44.3)	91 (42.3)	
Dyspnea, *n* (%)	22 (36.1)	27 (12.6)	<0.001
Upper respiratory tract infections in the previous 2 weeks, *n* (%)	33 (54.1)	116 (54)	
Diarrhea, *n* (%)	8 (13.1)	27 (12.6)	
Pericardial rub, *n* (%)	12 (19.7)	92 (42.8)	=0.001
Pleural effusion, *n* (%)	25 (41)	71 (33)	
Pericardial effusion, *n* (%)			
Mild/moderate	17 (28)	121 (56)	<0.001
Severe	44 (72.1)	0 (0)	<0.001
Cardiac tamponade, *n* (%)	11 (18)	0 (0)	<0.001
Emergency pericardiocentesis *n*, (%)	7 (11.4)	0 (0)	<0.001
ECG typical changes, *n* (%)	45 (73.8)	159 (74)	
WBC > 10^3^/mm^3^, *n* (%)	53 (86.9)	187 (87)	
CRP > 2.9 mg/L, *n* (%)	61 (100)	212 (98.6)	
cTnI > 0.045 ng/ml, *n* (%)	25 (41)	52 (24.2)	=0.012
ESR > 20 mm/h, *n* (%)	61 (100)	205 (95.3)	
Comorbidities^†^, *n* (%)	5 (8.1)	0 (0)	<0.001

Values are mean ± SD or *n* (%). ECG indicates electrocardiogram; WBC, white blood cells; CRP, C-reactive protein; cTnI, cardiac troponin I; ESR, erythrocyte sedimentation rate. ^*∗*^*p* value is reported in the table only when it was <0.05. ^†^Comorbidities were Hashimoto's disease, De Quervain's thyroiditis, and haemolytic autoimmune anemia.

**Table 4:** Follow-up data of the study population.

Feature	Total (276)	ASA/NSAIDs (215)	Prednisone (61)	*p* value
Recurrence, *n* (%)	171 (62)	137 (63.7)	34 (55.7)	0.257
Complete remission, *n* (%)	105 (38)	78 (36.3)	27 (44.3)	0.257

Values are *n* (%). The recurrences rate was not statistically different between the two groups.

**Table 5:** Follow-up data of the population with recurrent pericarditis.

Number of recurrences	1	2	3–4	5	Total
Patients, *n* (%)	109 (63.8)	43 (25.1)	18 (10.5)	1 (0.6)	171
Prednisone, *n* (%)	95 (55.6)	36 (21.1)	3 (1.8)	0	134 (78.4)
Prednisone + ASA/NSAIDs, *n* (%)	14 (8.3)	7 (4.1)	2 (1.1)	0	23 (13.4)
Prednisone + colchicine, *n* (%)	0	0	13 (7.6)	0	13 (7.6)
ASA/NSAIDs + colchicine, *n* (%)	0	0	0	0	0
IVIG, *n* (%)	0	0	0	1 (0.6)	1 (0.6)

Values are *n* (%). In the lines are listed the treatments of the recurrences and in the columns the *n*, % of patients who experienced the relative recurrence. IVIG indicates intravenous immunoglobulins.

## Data Availability

Our excel data are protected and we couldn't give it to anyone else on the basis of what our ethic committee established and of what the patients accepted in the informed consense.
